# Clinical Long-Term Response to Cardiac Resynchronization Therapy Is Independent of Persisting Echocardiographic Markers of Dyssynchrony

**DOI:** 10.14740/cr368w

**Published:** 2014-12-04

**Authors:** Barbara Naegeli, Hans-Peter Brunner-La Rocca, Christine Attenhofer Jost, Anja Fah-Gunz, Dominik Maurer, Osmund Bertel, Christoph Scharf

**Affiliations:** a HerzGefassZentrum, Klinik Im Park, Seestrasse 247, CH-8027 Zurich, Switzerland; b Department of Cardiology, Maastricht University Medical Centre, PO Box 5800, NL-6202 AZ Maastricht, The Netherlands

**Keywords:** Heart failure, Cardiac resynchronization therapy, Echocardiography, Doppler, Dyssynchrony, Outcome

## Abstract

**Background:**

The aim of the study was to prove the concept that correction of established parameters of dyssynchrony is a requirement for favorable long-term outcome in patients with cardiac resynchronization therapy (CRT), whereas patients with persisting dyssynchrony should have a less favorable response.

**Methods:**

After CRT implantation and optimization of dyssynchrony parameters, we evaluated whether correction or persistence of dyssynchrony predicted long-term outcome. Primary endpoint was a combination of cardiac mortality/heart transplantation and hospitalization due to worsening heart failure, and secondary endpoint was NYHA class.

**Results:**

One hundred twenty-eight consecutive patients (mean age 68 ± 10 years) undergoing CRT with a mean left ventricular ejection fraction of 27±9% were followed for 27 ± 19 months. All cause mortality was 17.2%, cardiac mortality was 7.8% and 3.1% had to undergo heart transplantation. Rehospitalization due to worsening heart failure was observed in 14.8%. NYHA class before CRT implantation was 2.8 ± 0.8 and improved during follow-up to 2.0 ± 0.8 (P < 0.001). A clinical response was observed in 76% (n = 97) and an echocardiographic response was documented in 66% (n = 85). After individually optimized AV and VV intervals with echocardiography, atrioventricular dyssynchrony was still present in 7.2%, interventricular dyssynchrony in 13.3% and intraventricular dyssynchrony in 16.4%. Despite persistent atrioventricular, interventricular and intraventricular dyssynchrony at long-term follow-up, the combined primary and secondary endpoints did not differ compared to the group without mechanical dyssynchrony (P = ns). QRS duration with biventricular stimulation did not differ between responders vs. nonresponders.

**Conclusion:**

After successful CRT implantation, clinical long-term response is independent of correction of dyssynchrony measured by echocardiographic parameters and QRS width.

## Introduction

Cardiac resynchronization therapy (CRT) has been successfully introduced into treatment of heart failure patients based on the concept that electromechanical abnormalities resulting from abnormal ventricular activation can be corrected by biventricular stimulation [1-4]. Whereas this therapy improved quality of life and functional status, reduced heart failure hospitalizations and prolonged survival in certain subsets of patients with prolonged QRS duration and reduced left ventricular ejection fraction (LVEF) [5-7], studies in other subgroups with moderate prolonged QRS duration and echocardiographic dyssynchrony were disappointing [8-10].

Even in patients with class I indications for CRT at least 30% do not respond to resynchronization therapy [6, 7]. We therefore studied whether long-term treatment effects are according to the intial concept dependent of correction of established echocardiographic and electrocardiographic parameters of dyssynchrony.

## Methods

### Patient population

All consecutive patients with successfully implanted CRT-P or CRT-D devices at our clinic as part of their clinical management in line with the current guidelines (LVEF ≤ 35%, QRS duration ≥ 120 ms, NYHA class II-IV despite optimal medical therapy) were recruited prospectively [11]. Both ischemic and non-ischemic cardiomyopathies were included. Medical therapy consisted of angiotensin-converting enzyme inhibitors or angiotensin receptor blockers, beta-blockers, diuretics and aldosterone antagonists as clinically tolerated and as deemed appropriate by the physician in charge.

### Study endpoints

Primary endpoint was definded as a combination of cardiovascular mortality/heart transplantation and/or hospitalization of worsening heart failure, and secondary endpoint was the change in NYHA class. Primary endpoint adjudication was performed by two experienced heart failure specialists blinded to echocardiographic and other follow-up data.

A clinical response to CRT was predefined as two of the following three criteria: decrease of NYHA class by at least 1 point, freedom of cardiac death within 6 months and freedom of rehospitalization for decompensated heart failure within 6 months. An echocardiographic response was predefined as an absolute increase in LVEF of ≥ 5% and/or a reduction of left ventricular end-systolic volume (LVESV) > 15% [12, 13].

### Echocardiographic acquisition

A complete standard transthoracic echocardiographic examination (Vivid 7, General Electric Medical Systems, Horton, Norway) was performed before device implantation. After successful CRT implantation, ventricular dyssynchrony was assessed with established echocardiographic techniques and tissue Doppler imaging (TDI) [12-15]. For each acquisition, three heart cycles were recorded. Doppler myocardial imaging velocity data were acquired using a narrow sector and optimal depth of imaging. The velocity range setting was adjusted in order to avoid aliasing.

Atrioventricular dyssynchrony was assessed by determining left ventricular filling time (LVFT), corrected for variations in different R-R intervals. A corrected LVFT of < 40% was used to indicate atrioventricular dyssynchrony [4]. Interventricular dyssynchrony was defined as an interventricular mechanical delay (IVMD) of > 40 ms and/or by a left ventricular preejection period (LVPEP) of > 140 ms [4]. Intraventricular dyssynchrony was defined as a septal-to-posterior wall motion delay (SPWMD) of ≥ 130 ms, and/or by a delayed activation of the lateral wall (DALW), which was calculated as a percentage of overlap between the end of lateral wall contraction on M-mode echocardiography and the onset of left ventricular filling [4, 6, 16].

The above mentioned parameters were collected in each patient to assess or exclude baseline ventricular dyssynchrony after device implantation (with nominal settings of the device). In patients with persistent ventricular dyssynchrony, individual optimization of atrioventricular (AV) and interventricular (VV) intervals was performed 1 month after device implantation by experienced echocardiographers in our heart failure clinic. Thus, AV interval optimization was done in over 90% with the iterative method, in the remaining patients with the simplified mitral inflow method or with the aortic velocity-time integral (VTI) or with the mitral inflow VTI method [17-19]. VV interval optimization was achieved by programming the settings in a way that would result in least VV mechanical delays (as measured by difference in pulmonary and aortic preejection intervals by pulsed-wave Doppler).

### Electrocardiographic measurements

Electrical conduction delays were measured on 12 lead electrocardiogram as QRS duration without biventricular stimulation and in biventricular paced rhythm, respectively.

### Study protocol

During long-term follow-up, all patients underwent clinical evaluation (NYHA class) and device interrogation before discharge, at 1, 3, 6 and 12 months, thereafter every 6 months. Under stable conditions, routine echocardiographic evaluation was performed every 6 months.

The study protocol was approved by the local ethical committee and all patients gave informed consent to participate in the study.

### Statistical methods

Categorical variables are presented as frequencies and percentages and compared between periods using the χ^2^ test. Continuous variables are presented as means ± standard deviation or medians with interquartile range (IQR) as appropriate and compared using Student’s *t*-test or Mann-Whitney U test as appropriate. Repeated measures were tested using paired *t*-test or Wilcoxon test as appropriate. All analyses were conducted using commercially available statistical software (SPSS version 18.0, SPSS Inc, Chicago, IL, USA). All P values are two-sided and considered statistically significant if ≤ 0.05.

## Results

A total of 128 consecutive patients (mean age 68 ± 10 years, 71% males) with successfully implanted CRT devices were included. One hundred fourteen (89%) were in sinusrhythm, 14 (11%) had permanent atrial fibrillation, and nine patients (7%) had an implanted pacemaker or ICD device prior to CRT implantation. Baseline charactersistics are shown in Table 1.

**Table 1:** Baseline Characteristics of the Included Population (n = 128)

Demographics	
Age, years	68 ± 10
Women, n (%)	37 (29)
Characteristics	
NYHA class	2.8 ± 0.8
Atrial fibrillation, n (%)	14 (11)
QRS duration (intrinsic), ms	162 ± 35
QRS duration (stimulated), ms	158 ± 28
AVB I, n (%)	30 (23)
LBBB, n (%)	88 (68)
LVEF, %	27 ± 9
LVEDD, cm	6.7 ± 0.9
LVESD, cm	5.6 ± 1.1
LVESV, mL	92 ± 0.7
Heart failure etiology, n (%)	
Idiopathic	59 (46)
Ischemic	47 (37)
Hypertensive heart disease	6 (5)
Valvular disease	12 (9)
Other	4 (3)
Comorbidities, n (%)	
Charlson score	2.7 ± 1.9
Hypertension	71 (55)
Diabetes	23 (18)
Hyperlipidemia	63 (49)
Positive family history for CAD	17 (13)
Smoker	70 (54)
Previous myocardial infarction	38 (30)
Previous PCI	31 (24)
Previous cardiac surgery	38 (30)
Medication, n (%)	
ACE inhibitor	70 (55)
Angiotensin receptor blocker	51 (40)
Beta-blocker	108 (84)
Diuretics	109 (85)
Spironolactone	66 (51)
Laboratory data	
Hemoglobin, g/L	135 ± 17
Creatinine, μmol/L	125 ± 77
BNP, ng/L	501 ± 649

AVB: atrioventricular block; LBBB: left bundle branch block; LVEF: left ventricular ejection fraction; LVEDD: left ventricular end-diastolic diameter; LVESD: left ventricular end-systolic diameter; CAD: coronary artery disease; PCI: percutaneous cardiac intervention; BNP: brain natriuretic peptide.

After a mean follow-up of 27 ± 19 months (range 2 - 98 months), all cause mortality was 17.2% (n = 22), cardiac mortality was 7.8% (n = 10) (eight low output and two sudden cardiac death) and 9.4% patients (n = 12) suffered a non-cardiac death (three cancer, four septicemia, two gastrointestinal bleeding, one cerebral hemorrage and two suicide). Four patients (3.1%) had to undergo heart transplantation (HTPL). The rehospitalization rate because of decompensated heart failure was 14.8% (n = 19).

In the entire group, functional NYHA class before CRT implantation was 2.8 ± 0.8 and improved during follow-up to 2.0 ± 0.8, LVEF increased from 27±9% to 37±13%, whereas left ventricular end-diastolic diameter (LVEDD) decreased from 6.7 ± 0.9 cm to 6.2 ± 1.0 cm and left ventricular end-systolic diameter (LVESD) decreased from 5.6 ± 1.1 cm to 5.0 ± 1.2 cm (all P < 0.001). NYHA class improved at least by 1 point in 57.8% (Table 2).

**Table 2:** Clinical and Echocardiographic Response After CRT Implantation in the Entire Group, n = 128 (%)

Clinical response	97 (76)
All cause mortality	22 (17.2)
Cardiac mortality	10 (7.8)
Non-cardiac mortality	12 (9.4)
Heart transplantation	4 (3.1)
Rehospitalization for decompensated HF	19 (14.8)
Decrease of NYHA ≥ 1	74 (57.8)
Echocardiographic response	85 (66)
Decrease of LVEF ≥ 5%	81 (63.3)
Increase of LVESV > 15%	50 (39.1)

HF: heart failure; LVEF: left ventricular ejection fraction; LVESV: left ventricular end-systolic volume.

Despite individual optimization of the AV and VV interval with echocardiography, atrioventricular dyssynchrony was still present in 7.2% (excluding 14 patients with permanent atrial fibrillation), interventricular dyssynchrony could be documented in 13.3% and intraventricular dyssynchrony was observed in 16.4%.

**Figure 1:**
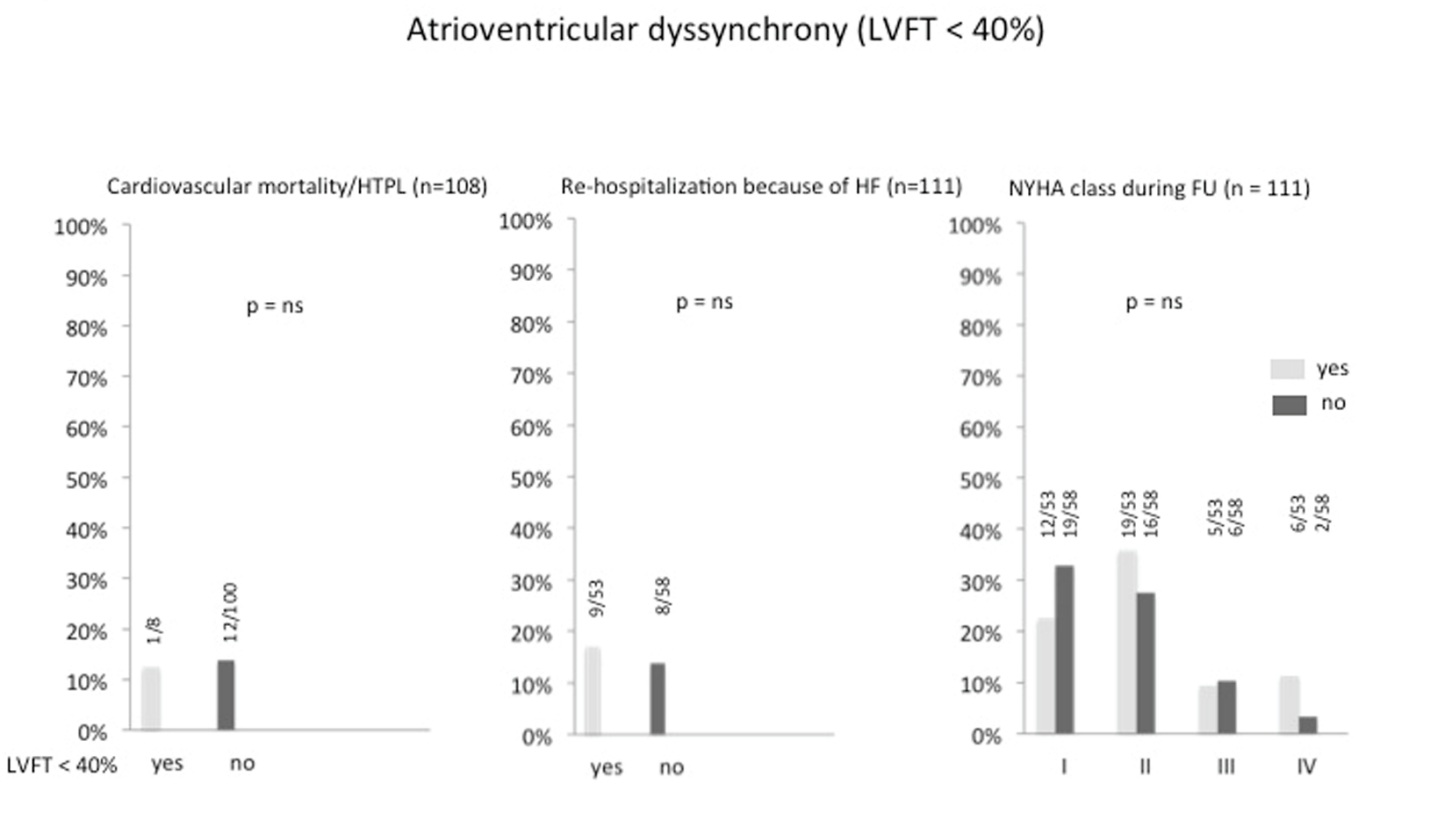
Percentage of atrioventricular dyssynchrony after optimizing the AV and VV intervals and its correlation to cardiac mortality/heart transplantation, re-hospitalization because of heart failure and NYHA class. HTPL: heart transplantation; HF: heart failure; LVFT: left ventricular filling time; FU: follow-up.

**Figure 2:**
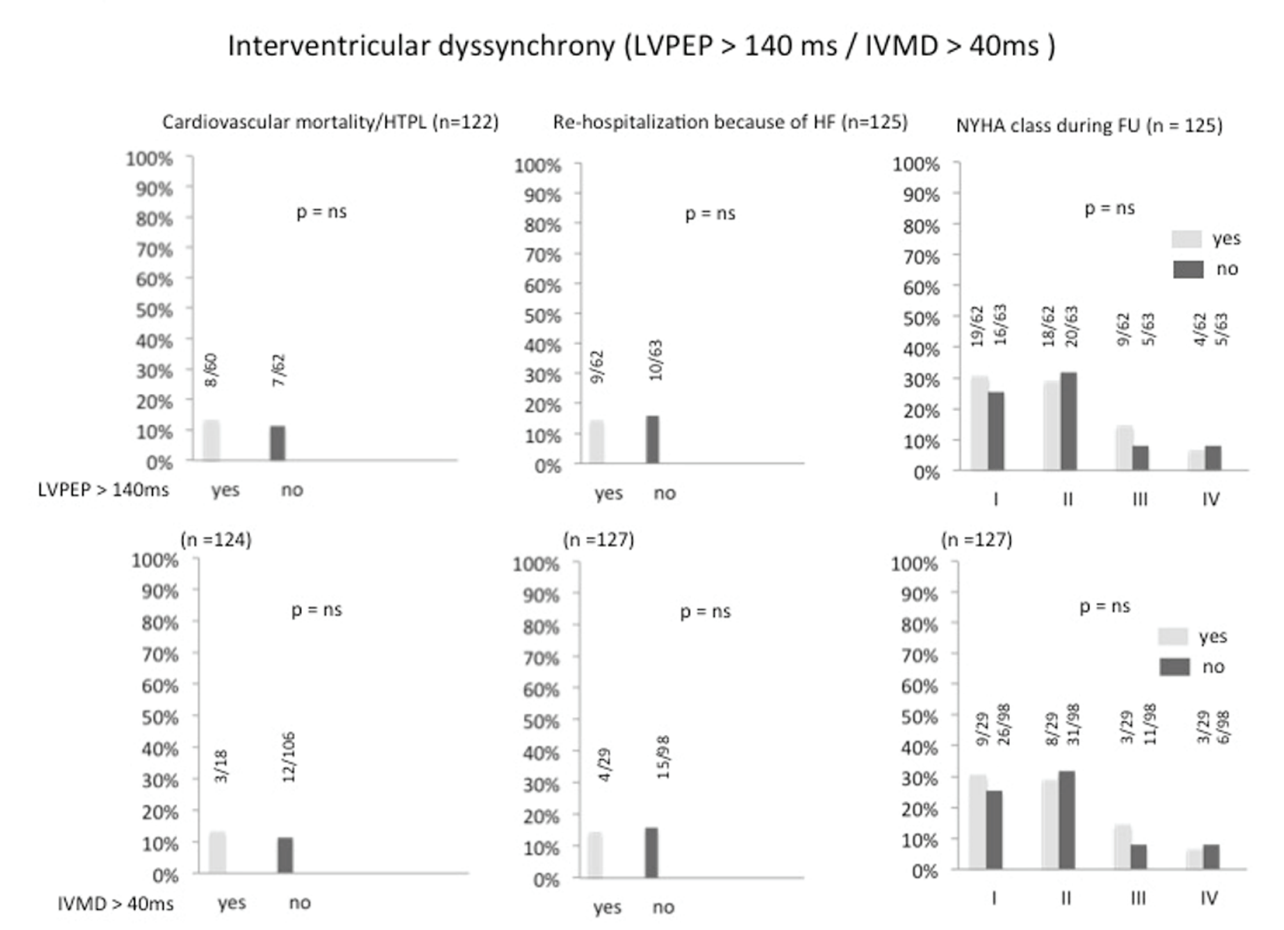
Percentage of interventricular dyssynchrony after optimizing the AV and VV intervals and its correlation to cardiac mortality/heart transplantation, re-hospitalization because of heart failure and NYHA class. HTPL: heart transplantation; HF: heart failure; LVPEP: left ventricular preejection period; IVMD: interventricular mechanical delay; FU: follow-up.

**Figure 3:**
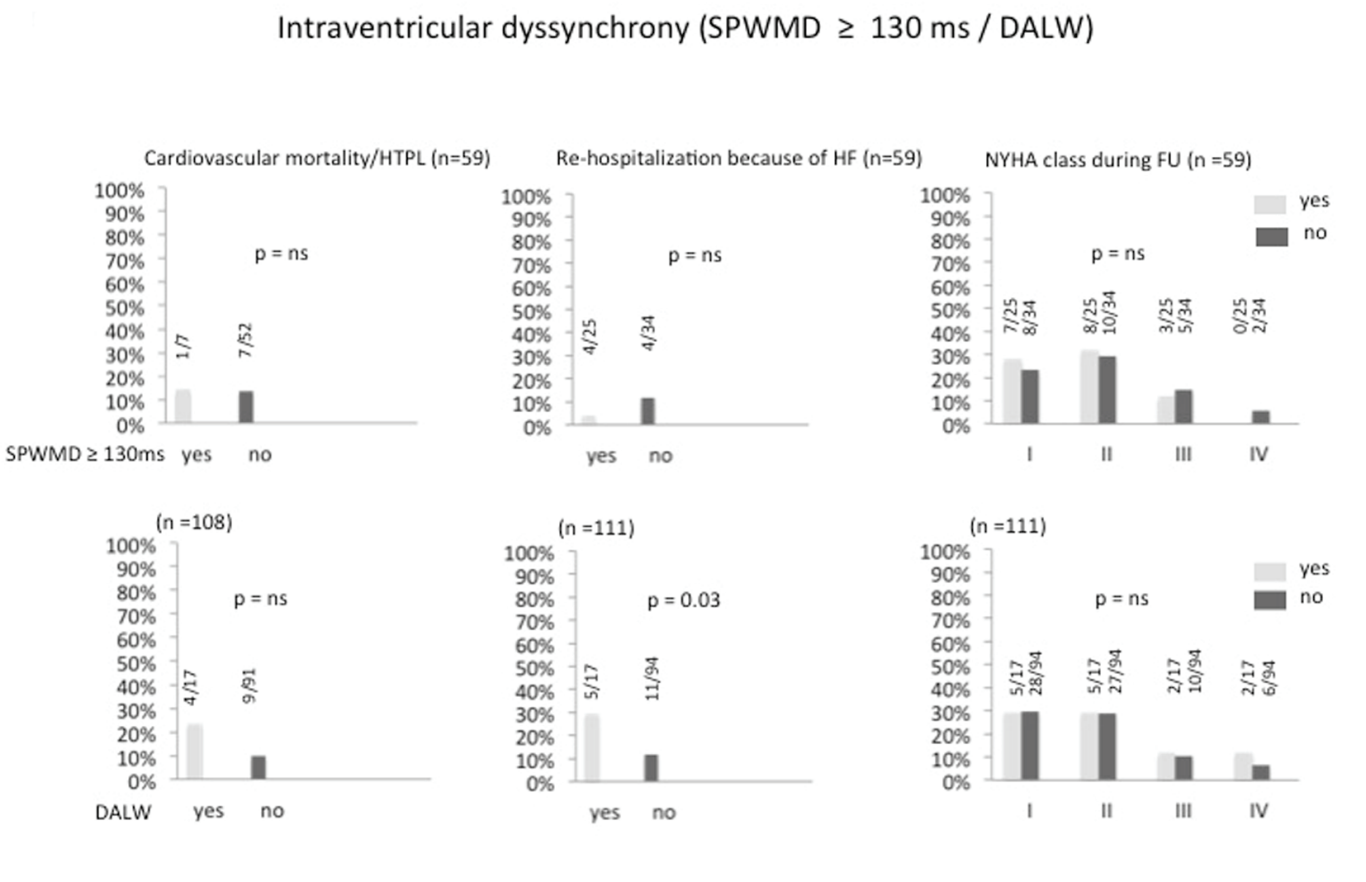
Percentage of intraventricular dyssynchrony after optimizing the AV and VV intervals and its correlation to cardiac mortality/heart transplantation, re-hospitalization because of heart failure and NYHA class. HTPL: heart transplantation; HF: heart failure; SPWMD: septal to posterior wall motion delay; DALW: delayed activation of the lateral wall; FU: follow-up.

At follow-up echocardiography, the combined primary and secondary endpoint did not differ between the groups with persistent or completely corrected atrioventricular dyssynchrony: cardiac mortality/heart transplantation and the frequency of rehospitalization due to progressive heart failure was equally distributed, and there was no difference in the distribution of functional NYHA classes between patients with and without atrioventricular dyssynchrony (Fig. 1). Likewise were the findings for persistent or absent interventricular dyssynchrony (Fig. 2) and for persistent or absent intraventricular dyssynchrony (Fig. 3).

Various clinical as well as echocardiographic parameters during follow-up were indicators of a poor outcome. Table 3 shows the difference in these parameters in patients with and without the combined endpoint of cardiac death and HTPL. However, none of the numerical echocardiographic dyssynchrony parameters were related to outcome and QRS duration after successful device implantation was also not predictive for outcome.

**Table 3:** Clinical, Echocardiographic and Electrocardiographic Characteristics Comparing Patients With and Without Cardiac Death or HTPL

	Survival wo HTPL	Cardiac death/HTPL	P
Age	69 ± 9	69 ± 14	0.96
Female gender, n (%)	35 (32)	2 (13)	0.15
CAD, n (%)	40 (36)	8 (53)	0.19
Diabetes, n (%)	16 (14)	6 (40)	0.02
Intial LVEF, %	27 ± 9	22 ± 7	0.03
Initial LVESD, cm	5.5 ± 1.0	6.3 ± 1.4	0.03
Initial NYHA class, median (IQR)	3 (2 - 3)	4 (3 - 4)	< 0.001
Initial QRS duration, ms	159 ± 33	177 ± 28	0.03
Biventricular stimulated QRS duration, ms	157 ± 28	170 ± 26	0.67
Systolic blood pressure, mm Hg	119 ± 16	101 ± 18	0.001
BNP prior to implant, median (IQR)	254 (133 - 515)	1,024 (374 - 1,803)	< 0.001
Creatinine prior to implant, median (IQR)	101 (84 - 128)	163 (99 - 224)	0.005
LVEF during FU, %	39 ± 12	25 ± 10	< 0.001
LVESV during FU, mL	62 ± 0.9	108 ± 1.4	0.009
NYHA class during FU, median (IQR)	2 (1 - 2)	4 (3 - 4)	< 0.001
Duration diastole (LVFT %)	50 ± 9	49 ± 8	0.46
LVPEP, ms	145 ± 34	152 ± 41	0.59
IVMD, ms, median (IQR)	16 (7 - 28)	17 (6 - 33)	0.61
SPWMD, ms, median (IQR)	60 (31 - 97)	70 (28 - 115)	0.83

wo HTPL: without heart transplantation; CAD: coronary artery disease; LVEF: left ventricular ejection fraction; LVESV: left ventricular end-systolic volume; IQR: interquartile range; BNP: brain natriuretic peptide; FU: follow-up; LVFT %: left ventricular filling time in %; LVPEP: left ventricular preejection period; IVMD: interventricular mechanical delay; SPWMD: septal-to-posterior wall motion delay.

## Discussion

Our study confirms that the clinical response with biventricular pacing was independent of successful or unsuccessful correction of established echocardiographic parameters of atrioventricular, interventricular and intraventricular dyssynchrony and was not related to shortening of QRS duration. Since the effects of CRT develop over several months and are thought to be due to ventricular reverse-remodeling, long-term follow-up is necessary to examine the relation between dyssynchrony and clinical response. The extended long-term observations in our study (2.3 years) are in accordance with the results of several other trials with shorter follow-up periods of 6 months to 1 year [20-23]. Given these results of clinical response being not related to established echocardiographic parameters of dyssynchrony, it is not surprising that virtually all trials failed which tried to improve clinical response by optimizing parameters of dyssynchrony either by modulation of AV or VV delays [20-23].

Equally prospective definition of responders vs. nonresponders using these echocardiographic parameters of dyssynchrony before device implantation failed, and only left bundle branch block as a reason for electrical conduction delay, clinical history and severly depressed LVEF remained powerful predictors of a positive treatment response [10]. There are newer attempts to define dyssynchrony and to better predict treatment failures by means of speckle tracking imaging and/or cardiac MRI [24-26]. One important potential of these methods is the identification of the site and extension of scars as well as extensive fibrosis, both of which have been shown to prevent an optimal treatment response to CRT [27, 28]. Whether these newer methods beyond that can better define responders versus nonresponders prospectively remains to be elucidated in long-term studies with larger patient cohorts.

In contrast to the lack of correlation between echocardiographic measurements of dyssynchrony and clinical response, we found several factors indicating poor outcome which are unrelated to cardiac dyssynchrony (Table 3). These included, not surprisingly, several markers of poor cardiac function, a finding which is in concordance with other studies showing that response to CRT is blunted in end-stage heart failure and suggesting the use of CRT implantation in earlier stages of the disease [7, 29]. This is also supported by recent randomized trials in less severe heart failure [7, 30]. On the other hand, diabetes and poor renal function were significantly related to poor outcome and lack of clinical improvement, highlighting the need of device studies in all-comers including patients with significant co-morbidities like in our study. These patients are poorly represented in prospective randomized trials. However, concurrent risks in severely comorbid patients may have a decisive role for outcome prediction on long term.

### Study limitations

A main limitation of our study is the heterogeneity of patients, including all patients with biventricular pacing implanted in our center according to current guidelines (all-comers). Therefore, patients with non-ischemic cardiomyopathy as well as patients with coronary artery disease encompassing myocardial scar areas (possibly preventing favorable responses) were included. A substantial part had comorbid conditions which may have influenced outcome predominantely. On the other hand, the study was not powered enough to perform subgroup analyses or to correct for these factors, e.g. by propensity score analyses.

A further limitation is the definition of “responders” to therapy, which, however, is inherent to all similar studies. We tried to overcome this weakness by assessment of the response by experienced specialists blinded for the echocardiographic and electrocardiographic follow-up measurements related to dyssynchrony.

### Clinical consequences and conclusions

In many institutions in-depth echocardiographic patient assessment is an integral part for optimal guidance of CRT. Repeated echocardiographic assessment of dyssynchrony and repeated reprogramming of the devices accordingly, is costly, technically challenging and time consuming. In accordance with the evidence from selected patient groups from randomized trials, our results on long term in unselected all-comers show that persistence or absence of dyssynchrony has no impact on outcome. Therefore, it seems reasonable to reduce markedly the complexity and frequency of echocardiographic follow-up examinations in patients with biventricular pacing.

More important, however, seems to be the consequence that the basic concept of “resynchronization” should be re-thought. It seems more appropriate to speak of modulation of dyssynchrony which is predominantely effective in patients with left bundle branch block and right ventricular pacing, whereas biventricular pacing to resynchronize patients with other forms of QRS prolongation or dyssynchrony with narrow QRS complexes is much more often futile.
